# Molecularly Imprinted Polymer-Based Sensor for Electrochemical Detection of Cortisol

**DOI:** 10.3390/bios12121090

**Published:** 2022-11-29

**Authors:** Elly Septia Yulianti, Siti Fauziyah Rahman, Yudan Whulanza

**Affiliations:** 1 Department of Electrical Engineering, Faculty of Engineering, Universitas Indonesia, Kampus UI Depok, Depok 16424, West Java, Indonesia; 2 Research Center for Biomedical Engineering, Faculty of Engineering, Universitas Indonesia, Kampus UI Depok, Depok 16424, West Java, Indonesia; 3 Department of Mechanical Engineering, Faculty of Engineering, Universitas Indonesia, Kampus UI Depok, Depok 16424, West Java, Indonesia

**Keywords:** antibody, aptamer, bulk imprinting, cortisol, electrode functionalization, molecularly imprinted polymer, surface imprinting, stress

## Abstract

As a steroid hormone, cortisol has a close relationship with the stress response, and therefore, can be used as a biomarker for early detection of stress. An electrochemical immunosensor is one of the most widely used methods to detect cortisol, with antibodies as its bioreceptor. Apart from conventional laboratory-based methods, the trend for cortisol detection has seemed to be exploiting antibodies and aptamers. Both can provide satisfactory performance with high selectivity and sensitivity, but they still face issues with their short shelf life. Molecularly imprinted polymers (MIPs) have been widely used to detect macro- and micro-molecules by forming artificial antibodies as bioreceptors. MIPs are an alternative to natural antibodies, which despite demonstrating high selectivity and a low degree of cross-reactivity, often also show a high sensitivity to the environment, leading to their denaturation. MIPs can be prepared with convenient and relatively affordable fabrication processes. They also have high durability in ambient conditions, a long shelf life, and the ability to detect cortisol molecules at a concentration as low as 2 ag/mL. By collecting data from the past five years, this review summarizes the antibody and aptamer-based amperometric sensors as well as the latest developments exploiting MIPs rather than antibodies. Lastly, factors that can improve MIPs performance and are expected to be developed in the future are also explained.

## 1. Introduction

Mental health issues have been heavily debated, especially during the COVID-19 pandemic, which affected various regions in each country as of early 2020. According to the World Health Organization (WHO), anxiety and depression disorders increased by 25% at the beginning of the COVID-19 pandemic [1]. Research conducted by Salari et al. revealed that the COVID-19 pandemic has increased the prevalence of anxiety, depression, and stress disorders by up to 35.3% [2]. Mental health disorders are triggered by multiple factors, but they most commonly begin with stress conditions that are not adequately managed and initiate chronic stress [3]. Not all stress conditions have visible symptoms, but when investigated molecularly, there are significant differences in physical conditions due to stress. Stress levels can be examined using physical functions, such as blood pressure, blood sugar, or heart rate. However, these examinations do not precisely indicate abnormalities in stress conditions. It was found that cortisol, the stress hormone, is a biomarker that strongly correlates with stress; this leads to physical function abnormalities that allow a rapid stress diagnosis.

Cortisol is a glucocorticoid hormone that is highly sensitive to all changes in body conditions due to internal and external disruptions. The conventional method using blood and urine collection was considered impractical [4], which triggered further research on other methods that can increase effectiveness and reduce pain during detection. In the last few decades, researchers have developed electrochemical cortisol detection methods by integrating bioreceptors (in the form of cortisol antibodies) through physical (adsorption and encapsulation) and chemical (cross-linking and covalent bonding) immobilization processes.

Detection using electrochemical methods provides various advantages, especially for manufacturing materials in biomedical applications. The electrochemical method requires minimum organic solvents and has a higher possibility in any electrode modification to amplify its performance in detecting various compounds, since it does not limit the complexity of the compounds it may detect. Therefore, biological samples are most suitable for this method by modifying the electrodes. The sensitivity, selectivity, and stability in detecting electroactive and non-electroactive compounds can be improved by integrating them with bioreceptors or other functionalization parameters [5]. This method can contribute to biomedical research because it is proven to be practical, is highly selective for cortisol molecules, works non-invasively, and can be monitored in real time [6].

However, antibody- and aptamer-based electrochemical detection is faced with obstacles, especially since antibodies are sensitive to temperature and ambient conditions [7]. Exclusive handling is required both during shipping and use, considering its high risk of denaturation [8]. The high price and short shelf life make the use of an antibody and aptamer as a bioreceptor in a wearable sensor impractical. Various studies have been carried out to overcome the limitations in determining reliable cortisol detection methods by prioritizing environmental stability and bioreceptor selectivity [6].

The molecularly imprinted polymer (MIP) demonstrates promise in improving cortisol detection quality. By manipulating the non-covalent interactions between monomers, molecular templates, cross-linkers, and solvents, MIPs can produce a polymer chain with pockets or cavities with shapes and functional groups corresponding to the template molecule. In this case, cortisol is used as the template molecule [9]. The MIP that has been formed will undergo a molecular template removal process to eliminate all the remaining cortisol for the rebinding process with cortisol in the actual sample [3]. The formed binding cavities corresponding to cortisol’s active site will develop a highly sensitive and specialized receptor to detect cortisol molecules in any samples tested [8]. It also achieves high repeatability since their interaction is non-covalent. MIPs are also well structured by polymer chains containing monomers and cross-linkers with a low possibility of degradation.

In this study, the MIP was more stable in ambient conditions and had a longer shelf life than antibodies and aptamers. MIP also has high specificity for molecular targets, works non-invasively, has relatively low fabrication costs, requires more straightforward handling, and is more applicable in future development [7]. MIPs have been developed as an electrochemical method since 2016. Its development grows as the public pays more attention to issues related to mental health. The molecularly imprinted polymer is an alternative bioreceptor for the electrochemical detection of cortisol.

This paper aims to highlight alternatives that can replace detection methods with antibody and aptamer bioreceptors sensitive to changes in environmental conditions. This paper will also provide recommendations for MIP methods that allow cortisol detection based on similar studies that have been carried out over the past few years.

## 2. Methods

This paper examined the development of cortisol detection via electrochemical immunosensors, both antibody- and aptamer-based, with a recently developed technique using molecularly imprinted polymers functionalized into the electrode. A literature study was carried out by collecting documents relevant to this topic from books, journals, and proceedings from the past five years. The data were collected through various references that grouped the subjects by their electrode modification process and MIP formation techniques.

## 3. Discussion

Cortisol is a steroid hormone with a metabolism that is closely related to the stress response. Fluctuations in the autonomic nervous system directed towards the adrenal glands influence how the body reacts to external danger. This reaction is called a fight-or-flight response [10,11]. These fluctuations affect the production of epinephrine and norepinephrine, which are related to blood sugar levels, immunity, and blood pressure [12]. The excessive fluctuations of these two compounds at an inappropriate time affect the stress conditions; cortisol is one of the hormones released with them [13].

Most of the cortisol in the body binds to serum albumin and globulin (corticosteroid-binding globulin), which transport cortisol through the cytoplasm to reach all analytes in the body. Internal cortisol concentrations range from 1.45 × 10^−6^ to 2.54 g/mL [3]. Its concentration in the blood, which is then excreted into the urine, is 25 × 10^−3^ g/mL in the morning and 2 × 10^−3^ g/mL at night [14], leaving 10% of free cortisol flowing through the rest of the analytes [15]. Recent research focuses on wearable sensors that promote a non-invasive detection system. As a result, saliva and sweat are commonly used analytes in most works. The typical concentration of saliva ranges from 10.2 to 27.3 × 10^−9^ g/mL in the morning and 2.2 to 4.1 × 10^−9^ g/mL at night [16]. Sweat has a typical range of 8.16 to 141.7 × 10^−9^ g/mL [17].

Several conventional and developing procedures are commonly used for cortisol detection. Conventional methods require laboratory-based instrumentation with expert handling, such as high-performance liquid chromatography (HPLC) [18,19,20,21], mass spectrometry (MS) [22,23,24], enzyme-linked immunosorbent assay (ELISA) [25], chemiluminescence immunoassay [26,27], and other methods with an insufficient intensity of use for cortisol detection, thus requiring continuous monitoring. The promising approach to overcome this problem is the electrochemical method. This method includes modifying the surface of the transducer so that it binds with the bioreceptors that correspond to cortisol. The electrochemical readout requires the number of electrons on the transducer’s surface, which indicates how much cortisol binds to the bioreceptor. The transducer surface is usually modified by immobilizing the bioreceptor onto the transducer surface.

Cyclic voltammetry (CV) is a method to determine the electrochemical response of the three electrodes used in a circuit by providing some potential range, allowing the current to flow along the path during the oxidation and reduction cycle while identifying the peak current response. This detection method is used to define whether a chemical reaction takes place, the reversibility of an electrode, the amount of electroactive material attached to the electrode, and its stability. CV is influenced by scan rate, surface area, and electrode structure [28].

Electrochemical impedance spectroscopy (EIS) is an electrochemical detection method used to determine biomolecular interactions at the electrode by measuring the electrical impedance and phase angle using sinusoidal potential or current excitation at the electrode. EIS measures the current generated by alternating current as a function of time. It has been widely carried out because it has high sensitivity, low energy requirement, uses a small number of samples, and can be performed with a limited amount of solution [29].

Differential pulse voltammetry (DPV) is a characterization method used to measure a current using potential amplitude pulses at the linear potential. DPV is generally used to measure the linear range and limit of detection (LOD) as a parameter in evaluating the performance of an electrode. Usually, the potential range chosen in the DPV characterization is one in which there is no faradaic reaction in that potential range. Current values over the potential range are measured, and the difference is recorded [30].

Square wave voltammetry (SWV) is an electrochemical detection method that achieves better sensitivity analysis results by producing a current difference between direct and reverse currents. This method can minimize noise from the background current, making it suitable for identifying responses for low concentrations, although it is rarely used in electrochemical detection on biosensors [31].

### 3.1. Cortisol Antibody

The cortisol antibody is the most widely used bioreceptor for cortisol detection. It is a protein produced by the immune system to respond to harmful compounds in the body. It is sensitive to changing conditions and corresponds to the compounds of interest. These properties demonstrate the potential for it to be functionalized as a biological sensor, called an immunosensor, since it performs low cross-reactivity. Cortisol, a biomarker representing stress conditions in the body, can be detected by antibodies that have an active site complementary to cortisol.

**Figure 1:**
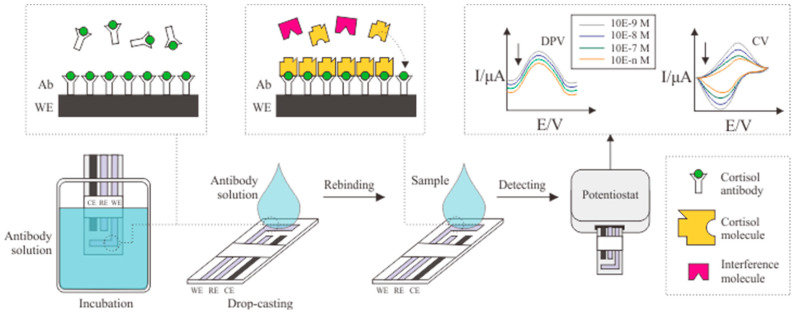
Schematic illustration of cortisol antibody immobilization in the electrode.

Several immobilization methods can be used to functionalize electrodes to bind with cortisol antibodies. Physical adsorption is one of the most widely used methods because no additional compounds are required to conduct the immobilization process. Figure 1 illustrates basic electrode modification using antibodies; the antibody and electrode surface interact electrostatically due to the isoelectric point difference between the electrode and antibody (IEP 4.50). The difference in IEP can produce covalent bonds [32,33]. This interaction can be carried out by drop casting the cortisol antibody solution [13,34] or electrode incubation in the cortisol antibody solution [35,36].

Additional to washing the remaining antibody with PBS, a study conducted by Nah et al. also used bovine serum albumin (BSA) to inactivate unbound antibodies. Soaking with a coupling agent such as 1-ethyl-3-(3-dimethylamino) propyl carbodiimide/N-hydroxy succinimide (EDC/NHS) was carried out after the immobilization process to strengthen the bond between the antibody and the electrode, which improves electrode surface stability [37].

This simple approach can detect cortisol with aa LOD of up to 5 × 10^−18^ g/mL. According to Sekar et al., this was obtained using hematite (α-Fe_2_O_3_), a type of nanostructured material with high stability integrated into a conductive carbon yarn (CCY) platform. The interaction of the intramolecular hydrogen bonds of these materials enhances the stability of CCY in the electrochemical readout [32].

Cross-linking is conducted by conditioning the electrode surface using a linker molecule to improve immobilized antibodies’ performance and affinity. Munje et al., Rice et al., and Kinnamon et al. have conducted experiments using cross-linking methods that yielded excellent electrochemical readout results with a LOD of 1 × 10^−9^ g/mL, which is low enough to detect cortisol in sweat, which has a minimum concentration of 8 × 10^−9^ g/mL. After incubating the electrodes in a solution containing a cross-linker such as dithiobis (succinimidyl propionate) (DSP) for 2–3 h, they are washed with PBS solvent. Washing was performed to remove unbound cross-linker molecules. The electrode cross-linker was incubated for 15 min in the cortisol antibody solution before being washed with PBS solvent [38,39]. Table 1 summarizes the difference between each modification of the electrochemical immunosensor in cortisol detection via antibody immobilization.

### 3.2. Aptamer

Aptamers also have characteristics that can be used for bioreceptor stress monitoring. An aptamer is a stable short-chain nucleic acid consisting of DNA or RNA that can identify small molecules, proteins, nucleic acids, or cells [42]. Aptamers have the advantages of a longer shelf-life, higher affinity for target molecules, greater resistance to denaturation, high stability, and more straightforward labeling [43]. The cortisol aptamer has the sequence GGAATGGATCCACATCCATGGATGGGCAATGCGGGGTGGAGAATGGTTGCCGCACTTCGGCTTCACTGCAGACTTGACGAAGCTT. The sequence partially depends on the type of modification and the purification process that is tailored to the design of each sensor. Some of the cortisol aptamers used in the formation of electrochemical immunosensors are modified with thiols (-SH) at 5′ [42,44,45,46], and amines (-NH_2_) and thiols at 5′ and 3′ [47,48,49], by the HPLC purification process. The immobilization process includes a cross-linker and physical adsorption through drop casting and incubation.

The electrochemical readout of the immunosensor with the aptamer-based bioreceptor is also sufficient, with a LOD of up to 47.12 × 10^−12^ g/mL, low enough to detect cortisol in sweat. Research conducted by Cantelli et al. involved utilizing the DNA superlattice integrated with gold nanoparticles to create a detection platform that can improve electron transfer and is more sensitive to target molecules. The DNA superlattice was obtained from the sequencing process between AuNPs and DNA to bind with the aptamer corresponding to the cortisol molecule. Operational parameters are required while applying a DNA-based linker. These parameters are largely influenced by the nature of the buffer, pH, and ionic strength [50].

Another successful method carried out on aptamer-based cortisol sensors is described in the study conducted by Huang et al., resulting in a low LOD of 0.09 × 10^−12^ g/mL. This study used MWCNT and mesoporous carbon material CMK-3, which are carbon nanomaterials that bond to each other through π–π interactions. Silver nanoparticles (AgNPs) are adsorbed onto composite nanomaterials through electrostatic interactions, enhancing electron transfer and signal amplification [47]. Table 2 recapitulates each modification of the electrochemical immunosensor in cortisol detection via aptamer immobilization.

It should be noted that the detection method using antibodies and aptamers can produce remarkably low detection ranges and LOD. It is necessary for immunosensor devices to be miniaturized in the future. This type of bioreceptor still has many obstacles, especially regarding its vulnerability to the surrounding environment. Although the fabrication process is relatively simple, antibodies and aptamers require a controlled environment for their fabrication. Aptamers and antibodies require further consideration before being developed into wearable sensors due to their relatively high cost and the risk of denaturation during shipping.

## 4. Molecularly Imprinted Polymer

A molecularly imprinted polymer is a type of polymer formed by covalent, non-covalent, or semi-covalent interactions between several monomers, cross-linkers, and solvents to form an artificial receptor system with antibody-like capabilities [8]. This type of bioreceptor has high environmental stability and a promising affinity to be considered an artificial antibody. The flexibility of MIP makes it an ideal foundation for the design of a bioreceptor to detect other macro-molecules and micro-molecules in the food, environment, and health sectors.

**Figure 2:**
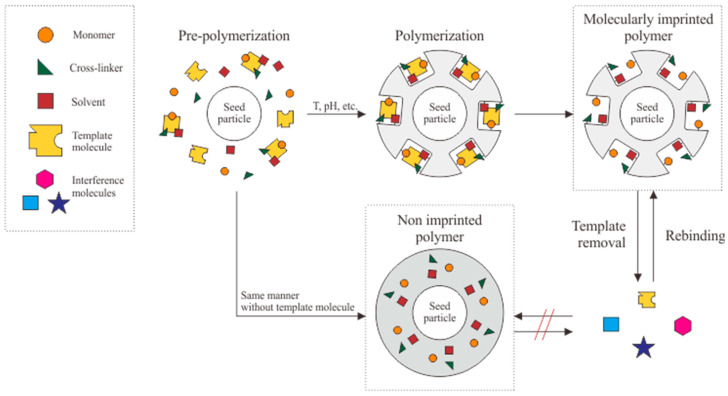
Schematic illustration of a basic molecularly imprinted polymer (MIP).

Non-covalent interactions are commonly studied as part of an imprinting method. Hydrogen bonds, ions, and van der Waals forces can be used as a medium to form appropriate stable binding cavities from the interaction between the formed polymer and the template molecule [51,52]. The non-covalent interactions also facilitate the template removal process for binding cavities formation and allow for multiple rebinding without any molecules remaining in the binding cavities [53]. Figure 2 demonstrates the basic formation process of a molecularly imprinted polymer.

Pre-polymerization, polymerization, and template removal are the three main phases in the MIP forming process. Pre-polymerization is the treatment used to initiate interactions between monomers, cross-linkers, solvents, and molecular templates. Polymerization is carried out by integrating monomer groups into polymer chains, which usually requires a longer duration. These two phases occur during the formation of binding pores or cavities, complementing the template molecule’s size and functional group [7]. Finally, template removal is carried out to eliminate all molecular templates used as the MIP mold. The template is removed via a washing process to allow the rebinding process.

As a control in evaluating the adsorption ability of MIP, a non-imprinted polymer (NIP) was formed with the same materials and methods but without template molecular addition. It is called NIP since the polymer chains formed on the transducer are the same. In this case, the resulting surface does not produce cavities, so the selectivity towards the target molecule is low. The imprinting factor (IF) is important for assessing the binding capacity of MIP versus NIP. The larger the IF ratio, the stronger the interaction between particles with a satisfactory affinity for the target molecule.

The IF ratio can be used to determine the best MIP formulation from the composition variation of the monomer, cross-linker, and solvent ratio. The ratio needs to be adjusted to prevent each compound function from overlapping. The monomer is used as the primary material and controls the surface area and MIP conductivity, which refers to the electron transfer activity on the movement of the target molecule [54]. The cross-linker or anchoring agent forms a bond between the electrode and the template molecule to produce a geometrically uniform MIP orientation. The choice of porogenic solvent is also vital in this process, because the solvent will influence the morphology and increase the surface area on the MIP surface to facilitate access both in the template removal and in the rebinding process with the target molecule [3]. Prioritizing the type of compound and its composition will cause the MIP to have complementary bonds and form selective binding cavities.

The resulting MIP is characterized by a scanning electron microscope (SEM). The structure and size of the MIP are determined under the microscope. Atomic force microscopy (AFM) can be used to characterize the polymer structure of the MIP due to the interaction of several constituent components [51]. The MIP is electrochemically characterized by cyclic voltammetry (CV), direct pulse voltammetry (DPV), or electrochemical impedance spectroscopy (EIS), or all three combined, to evaluate its ability to rebind the target molecule. This method entails utilizing the amperometric principle, where the sensor formed will be energized with the potential to generate currents. This is enabled by the oxidation and reduction processes of electroactive compounds. A redox probe in the form of ferrocyanide–ferricyanide is commonly used. This method can be used for the characterization process by scanning the magnitude of the peak current.

In addition to determining the sensitivity, specificity, linear range, and limit of detection (LOD) of the MIP, the electrochemical characterization process can also be used to monitor phenomena that occur during the formation process before the MIP is detected. In the case of cortisol MIP formation, the decrease in the oxidative and reductive peak current signal at each cycle of the electro-polymerization process indicates the formation of a polymer layer. The MIP formation is successful in this case. The increase in oxidative and reductive peak current signal with each cycle of the elution or template removal process indicates that more cortisol is removed from the MIP.

There are multiple modifications to enhance the performance of the MIP. Based on these findings, there are two types of MIP formation methods: bulk imprinting and surface imprinting. They differ significantly in process and final performance, which will be further explained in the next section.

### 4.1. Bulk Imprinting

**Figure 3:**
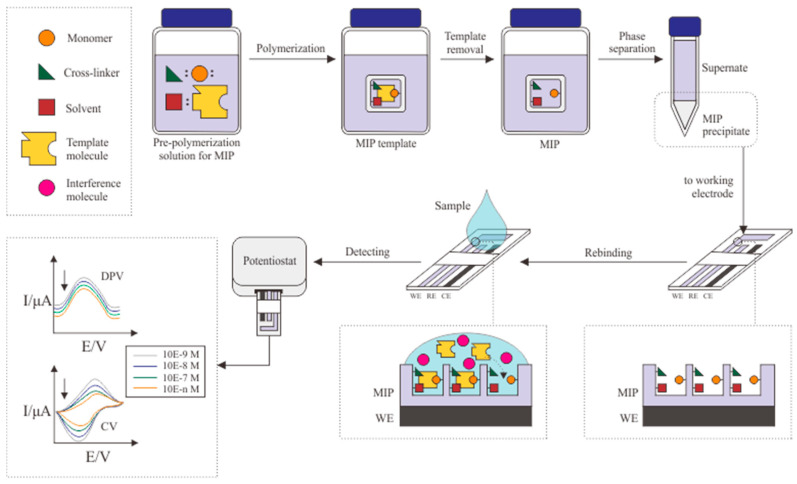
Schematic illustration of bulk imprinting method.

The popularity of bulk imprinting for the MIP manufacturing process still exceeds that of other polymerization techniques. It allows the polymerization process to be carried out in large quantities in one manufacturing batch. A monomer, cross-linker, porogenic solvent, and molecular templates will be dissolved with an adjusted ratio as a pre-polymerization solution. Several procedures and conditions are needed to initiate the polymerization process. The bulk imprinting method significantly varies as a result. Figure 3 exhibits a summary of commonly used procedures in the bulk imprinting process.

#### 4.1.1. Thermal Polymerization

Thermal polymerization is prevalent in the polymerization process, considering its convenient fabrication process, which can be used to achieve large production quantities with limited instrumentation. Klangphukhiew et al. carried out the polymerization process by placing the pre-polymerization solution into a nitrogen furnace at a temperature of 70 °C for 24 h [55]. Incorporating nitrogen gas into the pre-polymerization process [9,48,56,57,58], or during the polymerization process [55,59,60], has been applied to the bulk imprinting method. It works as a degassing or purging agent to remove dissolved oxygen and eliminate compounds with low molecular weights that can potentially cause the constructed polymer to return to the monomer form [61,62]. In contrast to Klangphukhiew et al., Mugo and Alberkant have produced an MIP with a remarkably lower LOD at the same temperature without a nitrogen atmosphere and with a shorter duration (4 h) [7].

#### 4.1.2. Photopolymerization

Photopolymerization involves using ultraviolet light to induce chemical reactions between monomer molecules and cross-linkers. The method consists of emitting UV light with a wavelength of 365 nm [9,63] from a rotating UV reactor [3,9,56], or using a medium-pressure Hg lamp [25], to initiate the polymerization process. The rotating reactor is used in this procedure to continuously increase the contact between the molecule’s surface and the emitted UV light energy. The process can take 12–42 h at 4 °C or 6 h at 60 °C, depending on the type of monomer used and the expected output [63].

#### 4.1.3. Radical Polymerization

Radical polymerization or controlled radical polymerization is also a common method due to the availability of materials. It is called radical polymerization because the materials and reagents used are toxic and require further consideration for the safety of medical applications. This method enables the regulation of the activation and inactivation processes to regulate the polymerization pathway. This procedure is relatively diverse, with atomic transfer radical polymerization (ATRP) and reversible addition–fragmentation chain transfer (RAFT) [64] being the most popular. They require a longer duration due to the pre-polymerization of intermolecular bonds resulting from intermolecular interaction. The method yields better morphology and density of the MIP’s inner layer [65,66]. So far, the RAFT method has not been utilized in the MIP cortisol application. As for the ATRP process, Suda et al. added a homogeneous MIP layer around the transducer by utilizing electron transfer as an activator; the polymer formation could then be carried out even at room temperature and when the molecular weight is unknown [57,67].

#### 4.1.4. Free Radical Polymerization

In contrast to radical polymerization, free radical polymerization prioritizes utilizing greener reagents and processes to generate eco-friendly by-products from polymerization residues. One exceptional finding regarding the bulk imprinting free radical polymerization method is the Ouzo polymerization process introduced by Kempe and Kempe. Ouzo is taken from the Greek alcoholic drink, made from essential oil and alcohol. The essential oil nucleates into white droplets when it is poured into the alcohol. Inspired by this observation, Ouzo polymerization is carried out without stirring and heating. The method centers on a spontaneous reaction between the monomer and solvent. It also results from preparing a composition with a phase diagram where the monomer nucleates and transforms into a solid polymer. The Ouzo polymerization parameter is referred to as the green procedure by the Rowan solvent greenness index (RSGI) of the overall solvent index (OSI) calculation. Based on these index calculations, 1 kg of MIP resulting from Ouzo polymerization has an RSGI value of 187. Most polymerization methods have an RSGI value above 250 [58].

Template removal is carried out after the polymerization method to remove the template molecule from the MIP surface. This process also varies, and can include Soxhlet extraction [56,60,68], sonication with organic solvents [9], centrifugation [58], or washing [3,7,25,55,57,59,69]. The process selection is based on the polymer phase because some MIPs are not formed on the surface of the transducer but in separate batches [25,58]. Daniels et al. have produced MIPs in the form of a powder that must be dissolved with anhydrous methanol to form a suspension to initiate template removal through centrifugation [9]. Table 3 presents the details of each publication on MIP formation via bulk polymerization.

So far, the method with the lowest LOD is radical polymerization by Usha et al., with an LOD of 25.9 × 10^−15^ g/mL, in which pyrrole polymerization is initiated and accelerated by adding metal oxides. In this case, zinc oxide (ZnO) plays a vital role in increasing the conductivity of polypyrrole from its random arrangement and increasing the mobility of electron transfer during electrochemical readout. ZnO also produces good results with lossy mode resonance (LMR) because it is biocompatible, inert, has a high isoelectric point (9.5), and has other beneficial physical and chemical characteristics [69].

Bulk imprinting has advantages that makes this method rather promising for large-scale production in the future. This method can be modified by various polymerization techniques. It can also include the modification of various pre-polymerization compounds. There are still concerns regarding micro-molecules, such as cortisol, due to the fragile nature of such molecules. Its chemical structure is sensitive to temperature and environmental changes during polymerization. Several bulk imprinting techniques that use high temperatures will increase the potential for damaging the cortisol structure, which leads to binding cavities that are not homogenous and will reduce the selectivity of MIP.

The binding cavities on the MIP surface have different orientations. The polymerization process and the chemical structure of monomers, cross-linkers, solvents, and irregular template molecules (i.e., continuous stirring) cause different orientations of binding cavities and allow them to become trapped under the MIP surface. These trapped cavities interrupt template removal, rebinding, and electrochemical readout processes. The non-homogenous shape, size, and orientation of the cavity allows micro-molecules to rebind to the MIP cavity and cause cross-reactivity, reducing its selectivity.

### 4.2. Surface Imprinting

Surface imprinting is a method that utilizes the formation of binding cavities on the MIP surface, as opposed to bulk imprinting, which forms cavities on all sides and under the MIP surface. Surface imprinting can be conducted by electro-polymerization, which uses the principle of electron transfer by depositing monomers onto the transducer through an electrolyte medium. This method does not employ heat (thermal polymerization) or UV light (photopolymerization) to create complete polymer chains. The process is similar to that of the cyclic voltammetry analysis. It involves preparing three electrode systems and a solution containing a monomer, molecular template, porogenic solvent, and cross-linker. It consists of three steps of MIP formation processes, namely, polymerization, template removal, and MIP analysis, when rebinding with cortisol samples.

Pre-polymerization is similar to bulk imprinting. It involves preparing the solution, optimum conditions, and electrode functionalization if required. The polymerization process begins with the preparation of a solution containing monomers, molecular templates, porogenic solvents, and cross-linkers. Pyrrole was the most frequently used monomer for the MIP cortisol production process [70,71,72,73], followed by o-phenylenediamine [4,74]. Pyrrole is considered an appropriate monomer because it is conductive, can be synthesized in a liquid medium, and is chemically stable, especially at neutral pH. Pyrrole can be polymerized at overoxidation potential. It has a high affinity for cortisol binding sites and transducers [75].

Dykstra et al. mentioned that pyrrole has a stronger bond with cortisol compared to other steroid hormones (progesterone, estrogen, estradiol, etc.) [73]. Several other monomers also succeed in electro-polymerization and bulk polymerization. Acrylamide produced MIP with a LOD of 0.05 × 10^−9^ g/mL [54]. In 2019, it was mentioned that aniline could detect various steroid hormones with a remarkably low LOD, whereas cortisol reaches up to 2 × 10^−18^ g/mL. The low LOD is due to the formation of poly(ANI-co-MSAN) from cortisol reversibility, which allows the presence of carboxylic acid as a provider of H^+^ ions during polymerization. In addition, lower pH can increase the formation of polyaniline, which corresponds to the acidic pH of cortisol (6.03), and allows increased conductivity of the resulting MIP [76]. Compared to bulk polymerization, the variety of monomers used in electro-polymerization in MIP cortisol formation is still limited. It is also stated by Scheller et al. that the exploration of a potential monomer for the electro-polymerization process needs to be studied further [77]. Table 4 summarizes all modifications that has been carried out in electro-polymerization procedures.

**Figure 4:**
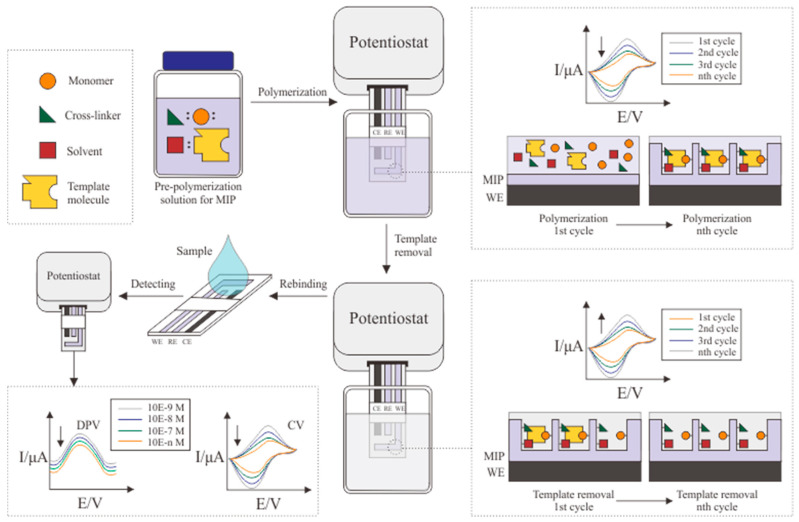
Schematic illustration of MIP formation via surface imprinting.

The additional components needed to generate MIPs are porogenic solvents. The selection of porogenic solvents for the electro-polymerization process is also still limited to phosphate-buffered saline (PBS) solutions suitable for biological research and capable of maintaining the properties of the dissolved molecules [78]. Although the bulk polymerization process often uses organic solvents such as chloroform, acetonitrile, dichloromethane, and methanol, the porogenic solvent still has limitations in the electro-polymerization process. There are also limitations regarding the template removal process in the electro-polymerization method, during which prolonged exposure to organic solvents is avoided, and PBS is used as a template removal agent. Research conducted by Gillan et al. demonstrated that organic solvents could cause swelling and damage to the MIP cavity due to prolonged immersion [71]. Figure 4 displays procedures in MIP formation via surface imprinting by applying cyclic voltammetry.

During the polymerization process, there is an interaction between cortisol and the monomer, resulting in an extensive binding affinity. At the molecular level, hydrogen bonds are formed by the oxygen atom interaction in the carboxyl group of cortisol with the nitrogen atom of pyrrole and the oxygen atom interaction in the hydroxyl group of cortisol with the nitrogen atom of pyrrole [71].

This method involves determining the optimal MIP thickness for each formulation ratio and adjusting the desired selectivity by controlling the MIP thickness. By adjusting the ratio of MIP formulation, scan rate, and polymerization cycle, the response current on the CV curve can conclude the optimum conditions. The optimum conditions will produce MIP structure with a thickness that allows it to cover the entire chemical structure of the cortisol template. MIPs with an excessively thick layer will also interfere with the template removal process. An excessively thin layer lowers MIP selectivity because it binds with many micro-molecules. The simple process that is not affected by temperature makes cortisol as a template molecule more resistant to its environment and able to maintain its shape until the end of the polymerization process.

A common obstacle encountered in the electro-polymerization process is the limited number of monomers suitable for this process. Although electro-polymerization can regulate the thickness of the formed MIP layer, it does not necessarily make the binding cavities’ orientation uniform. These obstacles, which appear in several studies, provide MIP cavities with the potential to bind to similar micro-molecules, since they are considered as artificial antibodies. The molecules with similar chemical structures will possess high cross-reactivity to cortisol, resulting in an unspecific electrochemical readout while detecting the cortisol in a sample. Progesterone, ethynyl estradiol, megestrol, norethisterone, dexamethasone, and meprednisone are some of the cross-reactants for cortisol molecules; most of these are steroid hormones. During MIP production, the specificities of its cavities must be prioritized to minimize cross-reactivity in order to achieve the best MIP configuration [79].

One major way to overcome this is to conduct electrode functionalization. Electrode functionalization can be carried out either by immobilizing the molecular template on the electrode prior to polymerization [80] or by self-assembling a monolayer (SAM) of the cross-linker compound by arranging the molecular template matrix on the electrode to allow the formation of binding cavities that match the orientation of the molecular binding site [77].

## 5. MIP Optimization and Electrochemical Detection of Cortisol

Electrochemical detection of cortisol is carried out in the same manner for both natural and artificial antibody-based immunosensors using a molecularly imprinted polymer. Cyclic voltammetry (CV) is commonly used by applying the amperometric sensor principle, which is widely used in the development of biomedical sensors. This method is the simplest, and provides straightforward detection results. The CV method is also used for the electro-polymerization process in MIP formation. By adjusting potential ranges, scan rates, and the number of scans in CV parameters, any increase or decrease in the voltammogram of each scan can be observed. Its layer formation behavior can also be analyzed through this adjustment [74].

The findings of these observations will be used to optimize the remaining process in order to achieve the best possible result parameters for the development of MIPs. The parameters that are considered in the optimization process, especially in the electro-polymerization procedure, are the number of scans, scan rate, ratio of pre-polymerization compounds (template molecule: monomer: solvent), elution scan rate, elution scan cycles, and template removal agent. Theoretically, the larger the number of these parameters, the better the detection and more specific results. There are some circumstances where optimum conditions are required, such as a greater number of scans will result in non-selective MIP, where the MIP layer is too thick and will cause cortisol molecules to become trapped in the cavity formed. Too many elution scan cycles may reduce the optimization of the energy source, where the voltammogram will show a stagnant peak current; this indicates that there are no more cortisol molecules left in the cavity even when more elution scan cycles are applied.

For electrochemical detection, a three-electrode system is integrated by an electrolyte in the form of a solution containing a solvent and a cortisol compound at various concentrations. Its objective is to obtain a calibration curve before the formed MIP is used to detect cortisol in the actual sample. This calibration curve can be obtained from the resulting peak currents from detection using CV and DPV methods over relevant concentration ranges. Since cortisol is a non-electroactive compound, each addition to the concentration leads to a decrease in peak current response [4]. In this case, the detection method with EIS is only accomplished to compare the peak current response to each electrode modification, not the formation of a calibration curve. The ability of EIS to reveal significant differences rather than the analysis resulting from CV and DPV detection methods makes it a more in-depth analysis with the help of charge transfer resistance (R_ct_). The R_ct_ value, which is the opposite of the voltametric current, can indicate more significant results and improve the analysis of redox probe diffusion. This enhances the sensitivity determination capacity of EIS through the Nyquist plot [81].

The detection process can then be carried out either on the actual samples or artificial samples. The original sample was obtained from sweat, saliva, serum, and other body fluids. The artificial sample can be prepared by manipulating a solution containing electrolytes, template molecules, and cross-reactants that are formulated to be as close as possible to the actual sample composition [72]. There must be several pretreatment processes to separate the fluid and dissolved impurities using centrifugation [13,33].

The electrode will be incubated for some period to increase the amount of the transfer molecule in the MIP cavities. The electrode will then be immersed in a redox probe solution, and the selected detection method will be applied to analyze the phenomenon of electron transfer of ferrocyanide and ferricyanide ions to the MIP cavities. With the presence of cortisol molecules in the cavities, electron transfer will be blocked. The resulting peak current will decrease as the cortisol concentration increases.

## 6. Conclusions

This work consists of a summary regarding the process of detecting cortisol by electrochemical immunosensor throughout the years, and its uniqueness using a molecularly imprinted polymer. We can conclude from the discussion that the molecularly imprinted polymer is a promising finding. MIPs can be used to develop a simple detection method with high accuracy without using easily denatured antibodies. This method can be developed by forming artificial bioreceptors from conductive polymers that have better durability and are flexible enough to be modified into various template molecules. So far, detecting cortisol using MIP has been possible with a very low LOD that allows detection with minimal samples or miniaturized sensors. Its simple manufacturing process also makes it in high demand, especially its long shelf life and environmental stability. The active site of the MIP cavity, which complementary imprints the molecular template, also has high selectivity for cortisol.

There is still the requirement for further work to overcome the drawbacks of each method, including homogenizing the orientation of MIP’s binding cavities. Furthermore, it is necessary to continue to investigate into selecting and estimating the best ratio for each material to create a biocompatible material to support biomedical applications.

By functionalizing the electrode template molecule immobilization into the electrode via a self-assembled monolayer (SAM) prior to the polymerization process, it is expected that the binding cavities formed can produce a better orientation to increase MIP selectivity. The variety of materials used demands to be explored further, especially in the electro-polymerization method, considering that the monomer used is limited to pyrrole and o-phenylenediamine, while the solvent used is phosphate-buffered saline solution. In the future, this method can be mass developed and commercialized gradually as a reusable detection system that is easy to use since it is easily miniaturized.

## Figures and Tables

**Table 1:** Summary of electrochemical immunosensors functioning via cortisol antibody immobilization.

Electrode	Immobilization Method	Detection Method	Bioreceptor	Linear Range (g/mL)	LOD (g/mL)	Matrix	Ref.
AuNPs–MWCNTs–PDMS	Physical adsorption	CV	Cortisol monoclonal antibody	1 × 10^−15^ to 1 × 10^−6^	0.3 × 10^−15^	Sweat	[13]
Carbon yarn	Physical adsorption	DPV, CV	Cortisol monoclonal antibody	1 × 10^−15^ to 1 × 10^−6^	5 × 10^−18^	Sweat	[32]
ZnO nanorod yarn based	Physical adsorption	DPV, CV	Cortisol monoclonal antibody	1 × 10^−15^ to 1 × 10^−6^	0.45 × 10^−15^ CV and 0.098 × 10^−15^ DPV	Sweat	[33]
Graphene	Physical adsorption	DPV	Cortisol monoclonal antibody	0.43 to 50.2 × 10^−9^	0.08 × 10^−9^	Sweat	[34]
Au	Physical adsorption	CV, EIS	Cortisol antibody	1 × 10^−12^ to 1 × 10^−6^	1 × 10^−12^	Sweat	[35]
MnO_2_	Physical adsorption	EIS	Cortisol antibody	0.1 to 1500 × 10^−12^	0.023 × 10^−12^	Sweat	[36]
Laser-burned graphene	Physical adsorption	EIS	Cortisol antibody	0.01 to 100 × 10^−9^	3.88 × 10^−12^	Sweat	[37]
ZnO	Cross-linking (DSP)	EIS	Cortisol antibody	10 to 200 × 10^−9^	1 × 10^−9^	Sweat	[38]
Pd/MoS_2_	Cross-linking (DSP)	EIS	Cortisol antibody	1 to 500 × 10^−9^	1 × 10^−9^	Sweat	[39]
Au	Cross-linking (DSP)	CV	Cortisol antibody	1 × 10^−12^ to 100 × 10^−9^	-	Sweat	[40]
Carbon-AuNPs	Physical adsorption	DPV	Cortisol antibody	22 to 386 × 10^−12^	7.47 × 10^−12^	Sweat	[41]

AuNPs–MWCNTs–PDMS, gold nano particles–multi-walled carbon nanotubes–polydimethylsiloxane; Au, gold electrode; MnO_2_, manganese (IV) oxide; Pd/MoS_2_, palladium/molybdenum disulfide.

**Table 2:** Summary of electrochemical immunosensors functioning via cortisol aptamer immobilization.

Electrode	Immobilization Method	Detection Method	Bioreceptor	Linear Range (g/mL)	LOD (g/mL)	Matrix	Ref.
ZnO	Physical adsorption	EIS	Cortisol aptamer	1 to 256 × 10^−9^	1 × 10^−9^	Sweat	[42]
AuNPs	Physical adsorption	SWV	Cortisol aptamer	30 × 10^−12^ to 10 × 10^−6^	10 × 10^−12^	Serum and Saliva	[44]
AuNPs	Physical adsorption	LSPR	Cortisol aptamer	0.03 to 362 × 10^−9^	0.03 × 10^−9^	Saliva	[45]
Au	Physical adsorption	nF-EIS	Cortisol aptamer	1 to 256 × 10^−9^	-	Sweat	[46]
GCE– MWCNTs/CMK-3/AgNPs	Physical adsorption	DPV	Cortisol aptamer	0.1 × 10^−12^ to 10 × 10^−9^	0.09 × 10^−12^	Saliva	[47]
GCE–FG–N-CQDs	Physical adsorption	CV, DPV	Cortisol aptamer	0.3 × 10^−12^ to 0.03 × 10^−9^	0.1 × 10^−12^	Saliva	[48]
Au	Physical adsorption	SWV	Cortisol aptamer	0.05 to 100 × 10^−9^	0.05 × 10^−9^	Serum	[49]
AuNPs	Cross-linking (DNA-based superlattice)	EIS	Cortisol aptamer	181 to 3600 × 10^−12^	47.12 × 10^−12^	Saliva	[50]

GCE–MWCNTs/CMK-3/AgNPs, glassy carbon electrode–multi-wall carbon nanotubes/ordered mesoporous carbon/silver nanoparticles; GCE–FG–N-CQDs, glassy carbon electrode–functionalized graphene–nano-carbon quantum dots.

**Table 3:** Summary of MIP formation via bulk imprinting method.

Electrode	Detection Method	Polymer	Solvent	Polymerization Technique	Linear Range (g/mL)	LOD (g/mL)	Matrix	Ref.
PEDOT/PSS	CV	MAA–EDMA	DCM	Thermal polymerization	0.03 to 3.6 × 10^−6^	-	Sweat	[3]
CNC/CNT	CV	MAA-co-EGDMA	Acetonitrile–water	Thermal polymerization	6 to 60 × 10^−9^	2.0 × 10^−9^ g/mL ± 0.4 × 10^−9^	Sweat	[7]
-	UV Analysis	MAA–EGDMA	DCM	Photo polymerization	0.03 to 0.36 × 10^−6^	-	Sweat	[9]
-	-	MISA–Acrylamide	Chloroform	Photo-polymerization	2.5 to 20 × 10^−9^	1.02 × 10^−9^	Saliva	[25]
SPCE	CV	MAA–Acrylamide	Toluene	Multi-step swelling and polymerization	0.46 to 7.25 × 10^−9^	0.43 × 10^−9^	-	[55]
PLLA	DPV	MAA	Chloroform	Bulk polymerization	3.6 to 29 × 10^−6^	3.6 × 10^−6^	-	[56]
Gold-coated glass	-	MPC–MBAAm–dimethyl sulfoxide	DCM	SI-AGET ATRP	-	1.74 × 10^−12^	Sweat	[57]
-	UV Analysis	PETRA	Ethanol–water	Free radical polymerization	-	0.69 × 10^−6^	-	[58]
NPs	-	Itaconic acid–styrene–DVB	THF	Radical polymerization	-	29 × 10^−9^	-	[59]
Fe3O4	UV Analysis	Fe_3_O_4_@SiO_2_-C=C/EGDMA/AIBN	Acetonitrile	Magnetic polymerization	0.01 to 0.1 × 10^−6^	0.004 × 10^−6^	Sweat	[60]
Ag	Raman Spectroscopy	MAA–AIBN–EGDMA	Methanol–toluene–dodecanol	Photo polymerization	0.03 × 10^−6^ to 0.36 × 10^−3^	0.03 × 10^−6^	Sweat	[63]
ZnO	UV Analysis	Py	Ethanol	Radical polymerization	1 × 10^−12^ to 0.1 × 10^−6^	25.9 × 10^−15^	Saliva	[69]

PEDOT/PSS, poly(3,4-ethylenedioxythiophene)/polystyrene sulfonate; CNC/CNT, carbon nanotubes/cellulose nanocrystals; SPCE, screen-printed carbon electrode; PLLA, polylactic (L) acid; NPs, nanoparticles; Fe_3_O_4_, magnetite; Ag, silver electrode; ZnO, zinc oxide; MAA, methacrylic acid; EDMA, ethylene glycol dimethacrylate; EGDMA, ethylene glycol dimethacrylate; MISA, molecularly imprinted sorbent assay; MPC, 2-methacryloyloxyethyl phosphorylcholine; MBAAm, N,N-methylenebisacrylamide; PETRA, pentaerythritol triacrylate; IA, itaconic acid; DVB, divinylbenzene; SiO_2_, core–shell polysilicate; AIBN, 2,2-azobis(2-methylpropionitrile); Py, pyrrole; DCM, dichloromethane; THF, tetrahydrofuran.

**Table 4:** Summary of MIP formation via surface imprinting.

Electrode	Detection Method	Polymer	Solvent	Linear Range (g/mL)	LOD (g/mL)	Matrix	Ref.
Au@GCE	CV, SWV, EIS	o-PD	Acetate buffer solution	0.36 × 10^−12^ to 181 × 10^−9^	72.5 × 10^−15^	Sweat	[4]
SPCE	EIS	Fullerene–acrylamide	PBS	0.18 to 23 × 10^−9^	0.05 × 10^−9^	Sweat	[54]
SPCE	CV	Py	PBS	0.36 × 10^−12^ to 3.6 × 10^−6^	0.36 × 10^−12^	Sweat	[70]
SPCE	CV	Py-PB	PBS	0.1 × 10^−9^ to 10 × 10^−6^	0.06 × 10^−9^	Sweat	[71]
Ag/AgCl	CV	Py-PB	PBS	-	0.207 × 10^−9^	Sweat	[72]
SPCE	DPV	Py	PBS–KCl	-	3.6 × 10^−12^	-	[73]
GCE/NiNC-N-CNTs	DPV	o-PD	PBS	0.36 to 3 × 10^−9^	0.86 × 10^−15^	Sweat	[74]
SPCE	CV	ANI-co-metanilic acid	Water	1 to 1000 × 10^−18^	2 × 10^−18^	Urine	[76]

Au@GCE, gold-nanoparticle-doped glassy carbon electrode; Ag/AgCl, silver/silver chloride; GCE/NiNC-N-CNTs, glassy carbon electrode/nickel-nanocluster-loaded nitrogen-doped carbon nanotubes; o-PD, o-phenylenediamine; PB, Prussian blue; ANI, aniline.

## Data Availability

Not applicable.
